# An Integrated Microfabricated Chip with Double Functions as an Ion Source and Air Pump Based on LIGA Technology

**DOI:** 10.3390/s17010087

**Published:** 2017-01-04

**Authors:** Hua Li, Linxiu Jiang, Chaoqun Guo, Jianmin Zhu, Yongrong Jiang, Zhencheng Chen

**Affiliations:** 1 School of Life and Environmental Sciences, Guilin University of Electronic Technology, Guilin 541004, China; longxingzu@126.com (L.J.); woshizhu.123@163.com (C.G.); 15107429521@163.com (J.Z.); zhangwei@guet.edu.cn (Y.J.); 2 Guangxi Experiment Center of Information Science, Guilin 541004, China

**Keywords:** needle-to-cylinder, ionic wind, ion source, air pump, LIGA

## Abstract

The injection and ionization of volatile organic compounds (VOA) by an integrated chip is experimentally analyzed in this paper. The integrated chip consists of a needle-to-cylinder electrode mounting on the Polymethyl Methacrylate (PMMA) substrate. The needle-to-cylinder electrode is designed and fabricated by Lithographie, Galvanoformung and Abformung (LIGA) technology. In this paper, the needle is connected to a negative power supply of −5 kV and used as the cathode; the cylinder electrodes are composed of two arrays of cylinders and serve as the anode. The ionic wind is produced based on corona and glow discharges of needle-to-cylinder electrodes. The experimental setup is designed to observe the properties of the needle-to-cylinder discharge and prove its functions as an ion source and air pump. In summary, the main results are as follows: (1) the ionic wind velocity produced by the chip is about 0.79 m/s at an applied voltage of −3300 V; (2) acetic acid and ammonia water can be injected through the chip, which is proved by pH test paper; and (3) the current measured by a Faraday cup is about 10 pA for acetic acid and ammonia with an applied voltage of −3185 V. The integrated chip is promising for portable analytical instruments, such as ion mobility spectrometry (IMS), field asymmetric ion mobility spectrometry (FAIMS), and mass spectrometry (MS).

## 1. Introduction

For an analytical instrument, the ion source is a very important component. It can ionize neutral sample molecules and transform them into positive or negative ions, which can be detected by the following detectors [1,2,3]. In previous studies, the ion source was usually used under the condition of vacuum, for which a large gas device was needed but was not suitable for portable instruments. In 2004, desorption electro-spray ionization (DESI) was first reported by Cooks et al., which is operated at atmospheric pressure and with little or no pretreatment of the sample [4]. After that, many studies on ambient ion sources have been successfully achieved, such as dielectric barrier desorption ionization (DBDI), direct analysis in real-time (DART), atmospheric solids analysis probe (ASAP), low temperature plasma (LTP), desorption corona beam ionization (DCBI), microwave-induced plasma desorption/ionization source (MIPDI), and so on [5,6,7,8,9,10].

In previous studies on the ambient ion source, the asymmetric electrode for the ion source has attracted much attention for its simple structure and high ionization efficiency. Kun Liu et al. adopt a line-cylinder electrode to form corona and glow discharges and ionize the sample, and the mass spectrometry (MS) and Faraday cup experiments show that the line-cylinder ion source has a high efficiency and stable propertiess [11]. Abdel Rahman et al. designed and fabricated a spherical configuration ion source, where the cathode fall is used to accelerate ions [12]. Ding et al. reported a novel ambient ionization technique for mass spectrometry. It is a microfabricated glow discharge plasma (MFGDP), where He or Ar plasma can be generated by a direct current voltage [13].

Although many attempts have been made to realize the ambient ion source, there is still an important problem that needs to be resolved. In the above-mentioned studies, nitrogen, air, or argon are employed as the carrier gas for cooling the air discharge region and carry ions through the next detecting part. The external air supply equipment needs to supply the gas, which is not appropriate for the portable instruments [14,15,16].

The work presented herein describes a micro-integrated system chip with integrated functions of the ion source and air pump, and the ionic wind is used to produce ionization and supply the gas. 

## 2. Device Design and Fabrication

### 2.1. Design of the Integrated System 

In the former studies of our group, a needle-to-cylinder electrode was designed and the ionic wind was obtained. The needle is parallel to the cylinder in this structure [17,18,19]. However, it is not favorable to fabricate the chip by micro-electro-mechanical system (MEMS) technology. We then designed a new configuration in which the needle is vertical to the cylinder. 

The ionic wind induced by corona discharge is described by the Equation (1), named as Poisson’s equation, current density equation, and the Navier-Stokes equations, respectively [20]:

(1) $$\left\{ \begin{array}{l} \nabla^{2}V=-\frac{\rho }{\varepsilon_{0}}\\ J=\mu_{E}E\rho +U\rho -D\nabla \rho \\ \rho_{air}U\cdot \nabla U=-\nabla p+\mu \nabla^{2}U-\rho \nabla V \end{array}\right.$$

where V is discharge voltage between the needle and cylinder electrode, ρ is the space charge density, $\varepsilon_{0}$ is dielectric permittivity of free space, J is current density, $\mu_{E}$ is ion mobility coefficient, E is electric field intensity, U is velocity vector of airflow, D is diffusivity coefficient of ions, $\rho_{air}$ is the air density, p is the air pressure, and μ is the air dynamic viscosity. 

According to the Equation (1), three modules named as Electrostatics (es), Incompressible Navier-Stokes (ns), PDE, and Coefficient Form (c) are set up in the COMSOL software (The COMSOL Group, Stockholm, Sweden). The other parameters are shown below. The total dimensions of the computation domain are 20 mm × 16 mm. The needle with a radius of curvature r=50 μm and the cylinder with radius R=2 mm are regarded as the cathode and anode, respectively. The physical parameters used in the simulation are $\varepsilon_{0}=8.85\times 10^{-12}$ C/(V·m), $\mu_{E}=2.1\times 10^{-4}$ m^2^/(V·s), $D=5.3\times 10^{-5}$ m^2^/s, $\rho_{air}=1.23$ kg/m^3^, and $\mu =1.8\times 10^{-5}$ N·s/m^2^. The voltage applied between the needle and cylinder is 9100 V. Four types of configuration, including single needle-to-single cylinder, single bottom needle-to-double cylinders, single central needle-to-double cylinders, and double needles-to-double cylinders are simulated.

**Figure 1:**
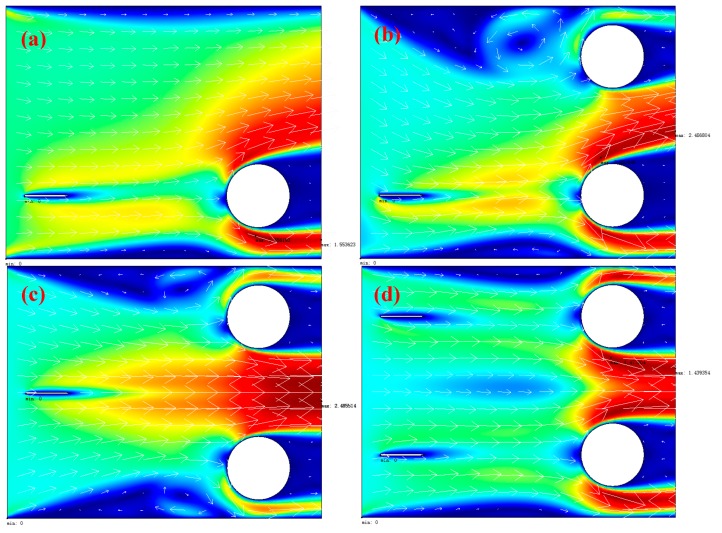
The Finite Element Method (FEM) simulation results for four different needle-to-cylinder structures: (**a**) single needle-to-single cylinder; (**b**) single bottom needle-to-double cylinders; (**c**) single central needle-to-double cylinders; (**d**) double needles-to-double cylinders.

The simulation results are shown in Figure 1. The ionic wind velocity for the single needle-to-single cylinder is about 1.55 m/s in the outlet. The wind velocity in the outlet for the single bottom needle-to-double cylinders and single central needle-to-double cylinders are almost the same at 2.49 m/s. The difference between them is that the ionic wind velocity for the single central needle-to-double cylinders is more parallel and uniform. However, the smallest velocity is 1.44 m/s for the double needles-to-double cylinders. The reason is that the ionic wind produced by the two needles will disturb each other. The similar phenomenon was also observed by the former studies [21]. According to the simulation results, it shows that the single central needle-to-double cylinder is the best structure in the four types of electrodes to get the maximum ionic wind velocity under the same conditions. 

**Figure 2:**
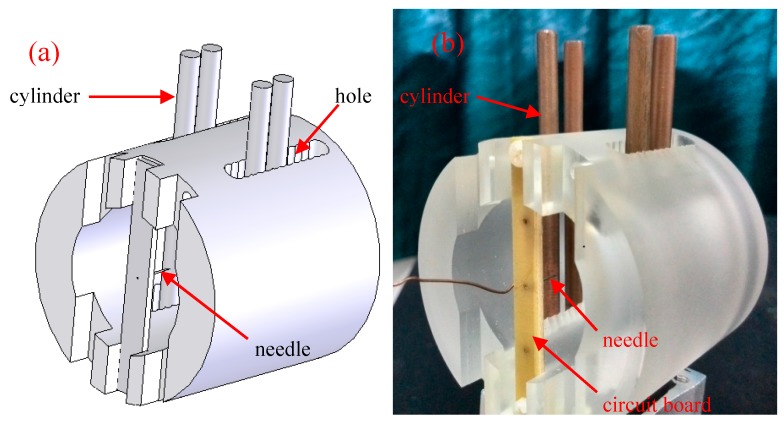
The schematic diagram of the experimental setup: (**a**) schematic diagram of the experimental setup; and (**b**) photograph of the experimental setup.

Based on the simulation results, the experimental setup shown in Figure 2 was designed and fabricated to observe the relationship between the cylinder number and ionic wind velocity of the systems. Discharge was produced between a needle and one or several cylinder electrodes. The needle had a sharp tip with a diameter of 27 μm, and the copper cylinder had a diameter of 4 mm and length of 60 mm. The distance between the needle and cylinder was adjusted by fixing the needle in different holes in the frame. The ionic wind velocity was measured with an anemometer (Testo 405-V1, Lenzkirch, Germany).

**Figure 3:**
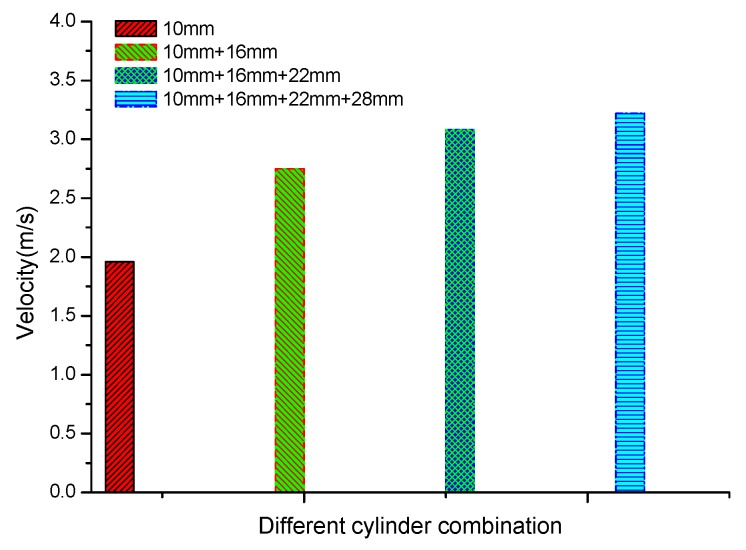
Dependence of ionic wind velocity on the groups of cylinders.

We conducted experiments using groups of different cylinder numbers with the applied voltage of −12 kV. Figure 3 shows that one group of cylinders with a gap distance *l* = 10 mm gave a small $\nu_{x}$ of 1.96 m/s. The combination of two and three groups of cylinders with *l* = 10 mm + 16 mm and *l* = 10 mm + 16 mm + 22 mm obtained $\nu_{x}$ of 2.75 m/s and 3.08 m/s, respectively. The maximum $\nu_{x}$ of 3.22 m/s was realized using a combination of four cylinders with *l* = 10 mm + 16 mm + 22 mm + 28 mm. The experiment shows that several groups of cylinders can obtain larger ionic wind velocities than that of one group of cylinders. The reason is that there exists air discharge between the needle and each group of cylinders. The ionic wind velocity is the mutual effect of each group of cylinders. Then the whole velocity is larger than that of fewer groups of cylinders.

**Figure 4:**
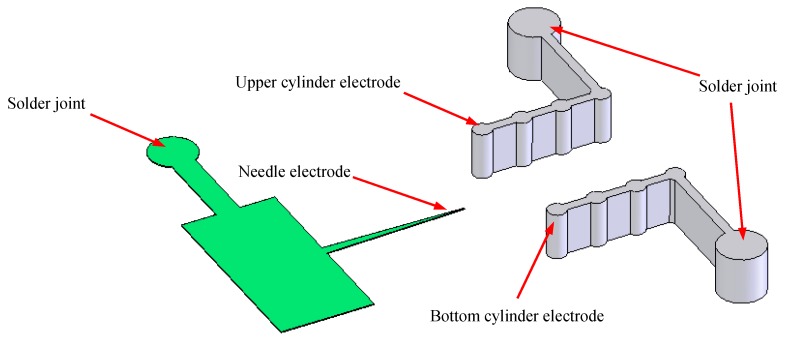
Design of the needle-to-cylinder electrode.

The above theoretical and experimental results show that the central needle-to-double cylinders structure and four groups of cylinder combinations are all favorable to increase the ionic wind velocity. To fabricate the group cylinders easily by Lithographie, Galvanoformung and Abformung (LIGA) technology, the cylinders are connected together. The schematic of the needle-to-double cylinders is shown in Figure 4. A needle with a tip diameter of 20 μm and thickness of 20 μm is employed as the discharge cathode. To generate stable air discharges, the anode is designed as a smooth cylinder. Compared with the anode of plate and mesh, the cylinder electrode more easily generates stable corona and glow discharges. Then a cylinder of 1 mm in thickness and 0.4 mm in diameter is used as the anode. To achieve the maximum ionic wind velocity and form an airflow channel, four cylinders with the same dimension are connected together, as shown in Figure 4. The gap distance between the upper cylinder electrode and the bottom cylinder electrode is 3 mm. The solder joints of the needle electrode and the cylinder electrode are designed in a circular shape with a diameter of 0.5 mm for the welding wire. 

**Figure 5:**
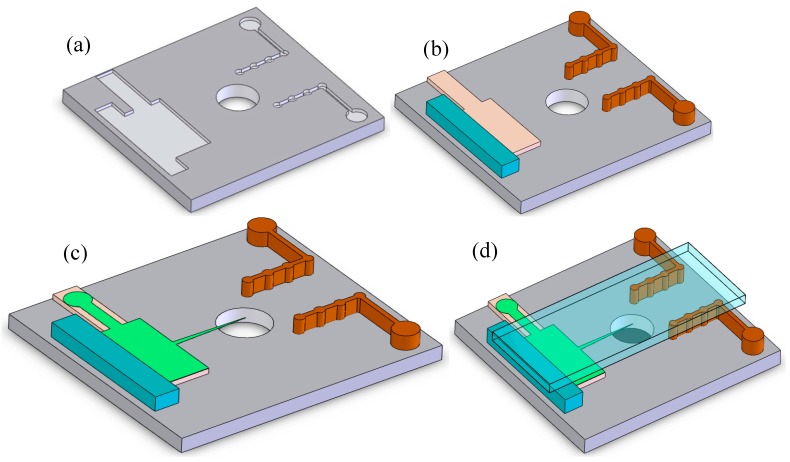
The steps of assembling the integrated chip: (**a**) the lower substrate; (**b**) mount the cylinders and the supports onto the lower substrate; (**c**) mount the needle onto the support; (**d**) place and glue the upper substrate.

The steps of assembling the integrated system are shown in Figure 5. Firstly, mounting holes are arranged on the lower substrate (Figure 5a). Secondly, the cylinder electrodes and the supports are mounted into the mounting holes (Figure 5b). Thirdly, the needle electrode is attached to the support (Figure 5c). Lastly, the upper substrate is manually assembled on the cylinder electrodes and the supports, and then the airflow channel is formed (Figure 5d).

The geometric dimensions of the chip are given in Table 1.

**Figure 6:**
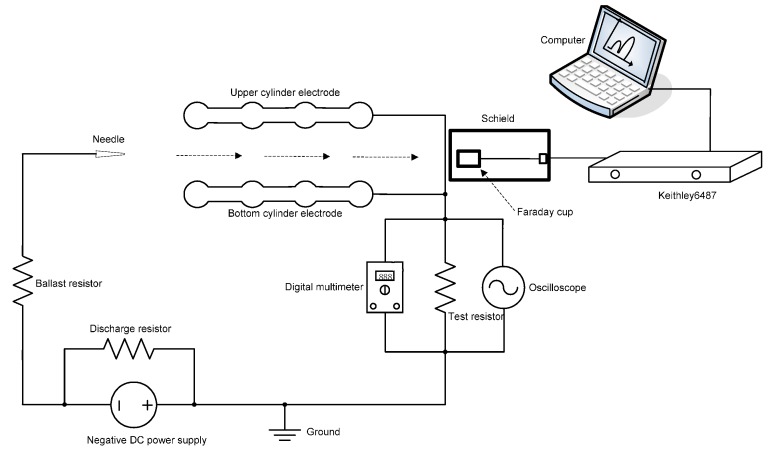
The schematic of the experimental setup.

When a high negative DC voltage is applied to the needle electrode and the two cylinders are connected to the ground, there will be an air discharge between the needle and the cylinders. Comparatively, the negative DC discharge is more stable than the positive DC discharge [22]. Then the discharge is powered by a 0 to −5000 V, 500 W DC power supply. The discharge circuit is shown in Figure 6. A discharge resistor of 12 MΩ is connected to the power supply to release the electric charge when the power supply voltage decreases. The needle is connected to the negative high voltage by way of a ballast resistor of 6 MΩ, which can restrict the discharge current. The upper and bottom cylinder electrodes are connected to the ground of the power supply by a test resistor of 1 kΩ. On the both sides of the test resistor, an oscilloscope (TektronixTDS1002B-SC, Beaverton, OR, USA) and a digital multimeter are used to record the actual AC voltage and the voltage effective value, respectively. The discharge images were recorded by using a high-resolutiondigital camera (Nikon D810, Tokyo, Japan) with a special micro lens. A Faraday cup is fitted into the chip outlet to catch ions ionized by the needle-to-cylinder electrodes. To eliminate interfere signal, the Faraday cup is enclosed in the metal shield. After that, the detected micro current is measured by a Keithley 6487 electrometer. The corresponding current data is stored in the computer by using LabVIEW software.

### 2.2. Material Choice

The integrated system is composed of three components bonded together: the needle and cylinder electrodes, the bottom substrate, and the upper cover. For the above three components, the main requirements are shown in the following:

(1) The needle-to-cylinder electrodes must be conductive materials and strong enough to resist the erosion of air discharge;
(2) The bottom and upper substrates should be an electrical insulator so as to accomplish the electrical isolation from the needle and the cylinders; and
(3) The upper substrate should be transparent to observe the discharging phenomenon.

According to the above requirements, electroplated Cu was selected as the material for the needle-to-cylinder electrodes. Although silicon is also conductive, its brittleness limits its use as the discharge electrode. Comparatively, electroplated Cu is not only conductive, but also has favorable mechanical properties and can resist the discharging erosion. Polymethyl Methacrylate (PMMA) was selected as the material of upper and bottom substrates for its good electrical insulation and transparent properties. 

### 2.3. Fabrication Process

**Figure 7:**
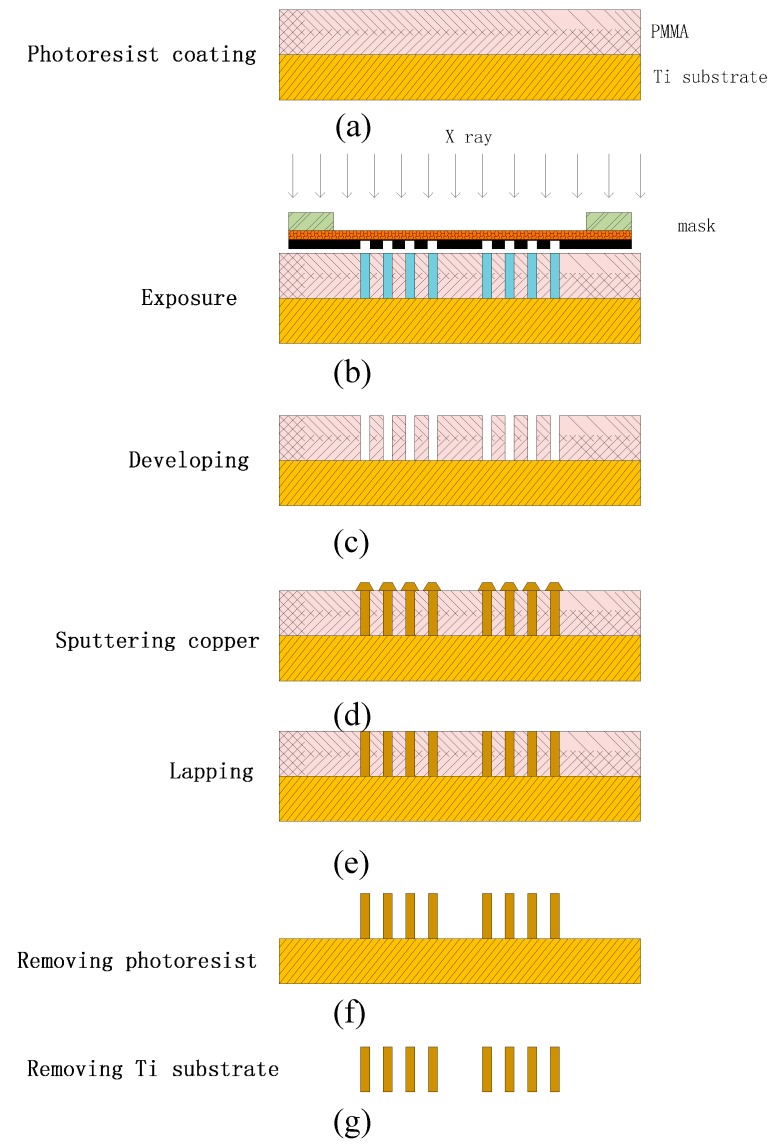
Fabrication details of the needle and cylinder electrode; (**a**) photoresist coating; (**b**) exposure; (**c**) developing; (**d**) sputtering copper; (**e**) lapping; (**f**)removing photoresist; (**g**) removing Ti substrate

The fabrication process for the micro cylinder electrode is shown in Figure 7. The chip is fabricated by LIGA technology. The main steps of the fabrication process are as follows:

(a) Photoresist coating: The photoresist is chosen as PMMA of AR-P 6510 style from ALLRESIST in Germany. The baking temperature is 160 °C lasting 50 min.
(b) Exposure: The time for the X-ray exposure is 2.2 h.
(c) Developing: The developing agent is R600-56, and the developing time is eight hours.
(d) Sputtering copper: The copper sulfate solution is used with the density of 200 g/L. The density of sulfate is 100 mL/L. The electroplating current is 200 mA at room temperature, and the lasting time under the electroplating current is 24 h. The height of electroplated Cu for the cylinder and the needle is 1 mm and 20 μm, respectively.
(e) Lapping: The grinding process is finished by a UNIPOL-802 grinding machine from MIT Company. The rotational speed is 50 r/min until the grinding process is finished.
(f) Removing the photoresist: The photoresist is removed by the Remover AR600-70.
(g) Removal of the Ti substrate: The Ti substrate is removed by 30% hydrofluoric acid with adensity of 20 mL/L for 8 h.

The fabrication process of the needle is similar to that of the copper cylinder, but there are still some differences for the substance. The photoresist is AZ5620 from AZ Company, prebaking at 90 °C for 40 min. The lithography process is realized by the BG-401A lithography machine, and the exposure time is five minutes. The development solution is 7% NaOH, lasting 10 min. The photoresist is removed by 20% NaOH. The electroplated solution is copper sulfate with adensity of 200 g/L, and the density of sulfate is 100 mL/L. The electroplated current is 200 mA at room temperature for 30 min. The thickness of the electroplated needle electrode is 20 μm. Finally, the Ti substrate is removed by 30% HF acid with adensity of 20 mL/L for four hours.

**Figure 8:**
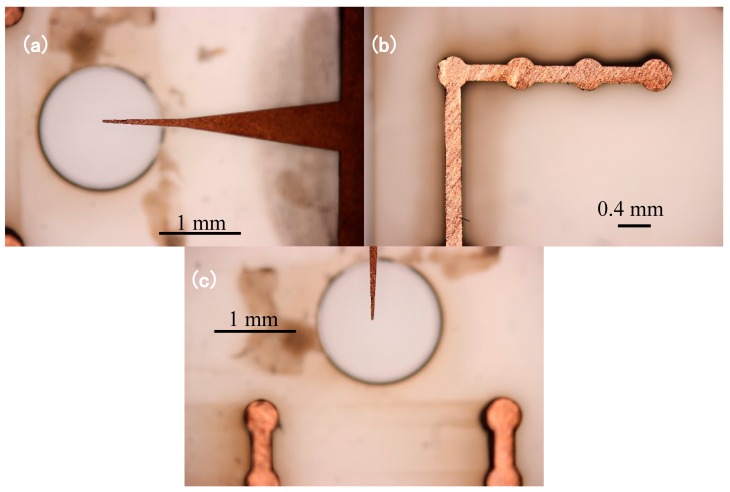
The needle and cylinder electrodes fabricated by Lithographie, Galvanoformung and Abformung (LIGA) technology: (**a**) needle electrode; (**b**) cylinder electrode; and (**c**) the assembled needle and the cylinder.

Figure 8 presents the needle-to-cylinder electrodes fabricated by LIGA technology. It shows that the needle is sharp-tipped. To increase the strength, the needle bottom is wider than the needle tip, and the needle tip is placed over the centre of the injection hole, which is designed to inject the sample substance. The cylinders are connected with each other by a 20 μm wide plate. Once assembled, the needle points to the center of the gap between the two cylinders (Figure 8c).

## 3. Results and Discussion

### 3.1. The Discharge and Ionic Wind Velocity

**Figure 9:**
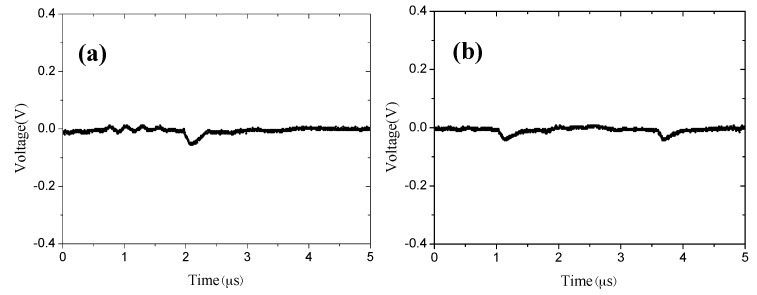

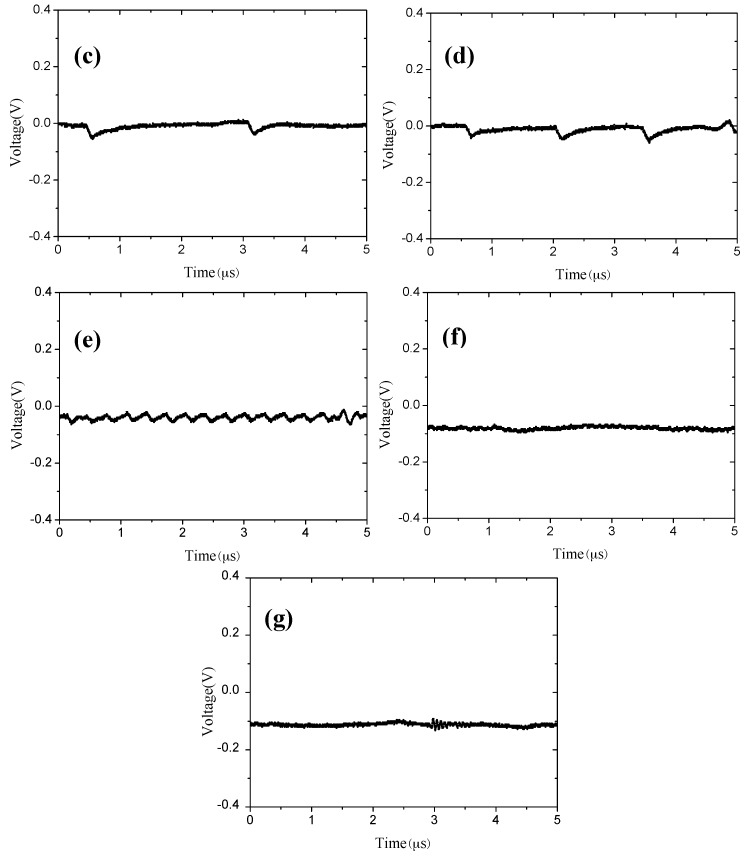
The chip discharge waveform with spacing of 2 mm at (**a**) −3050 V; (**b**) −3200 V; (**c**) −3300 V; (**d**) −3400 V; (**e**) −3500 V; (**f**) −3563 V; and (**g**) −3600 V.

The discharge waveform of the needle-to-cylinder electrodes was measured by the oscilloscope. At the beginning of discharge (with an applied voltage of −3050 V), there is a typical Trichel pulse (Figure 9a). As the Trichel pulse is the characteristic for corona discharge, this proves that the stable corona discharge is achieved by the needle-to-cylinder electrode without an external airflow [23]. With an increase in the applied voltage, the pulse frequency decreases correspondingly, and the amplitude of the pulse waveform also decreases. Then a DC component arises with an applied voltage of −3500 V (Figure 9e). At last, the Trichel turns into a DC line when the applied voltage is −3563 V (Figure 9f). This means that corona discharge transforms into glow discharge.

**Figure 10:**
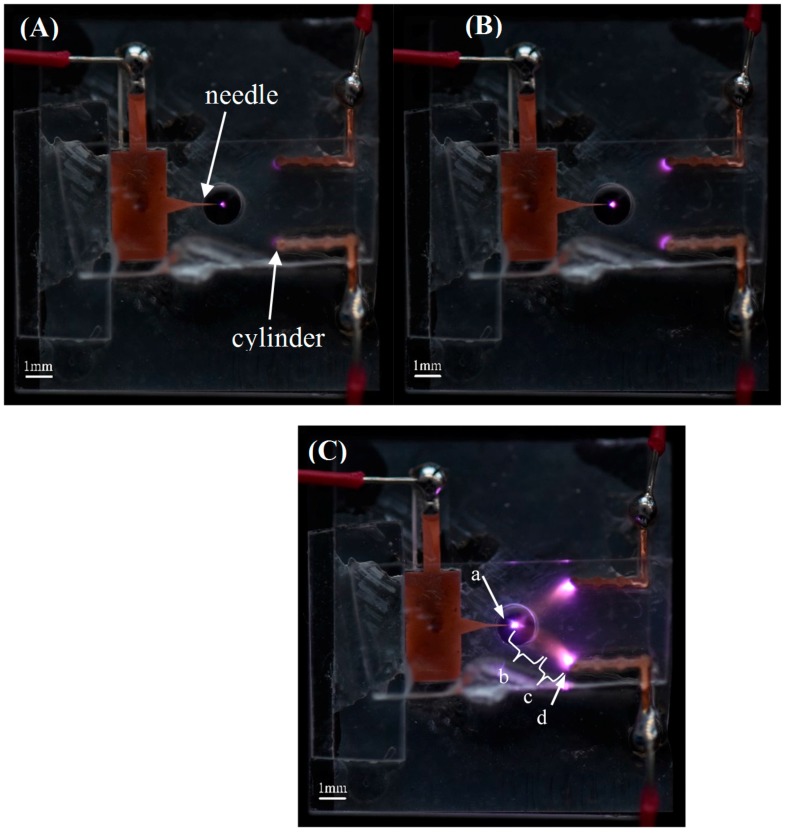
Images of the air discharge with different applied voltages (the gap distance d = 2 mm): (**A**) U = −3050 V; (**B**) U = −3500 V; and (**C**) U = −3600 V, (**a**) negative glow; (**b**) Faraday dark space; (**c**) positive column; (**d**) anode spot.

Figure 10 shows the chip discharge images recorded by a Nikon D810 camera. At the beginning of corona discharge (U = −3050 V, Figure 10A), the discharge light is mainly concentrated on the needle tip. Additionally, there is discharge plasma on the cylinder which faces the needle directly. However, on the other three cylinders, no discharge plasma is observed. With an increase in applied voltage (−3500 V), the discharge plasma becomes larger and brighter (Figure 10B). This corresponds to the transition stage from corona discharge to glow discharge. In previous work, the discharge plasma is mainly concentrated on the needle tip, and there was no, or little, discharge plasma on the cylinder surface [17,18,19]. A luminous plasma sheet appears between the electrodes. When the glow discharge is ignited (Figure 10C), there are familiar bright and dark regions shown in the low pressure glow discharge. The discharge space between the needle and the cylinder can be divided into four regions: negative glow, Faraday dark space, positive column, and anode glow. Near the needle tip, there exists the negative glow region which is a hierarchical structure. Following the negative glow, the Faraday dark space appears. In this region, there is no apparent brightness. It can be found that moving from the ‘Faraday dark space’ there is a ‘positive column’ region. In this region, the bright discharge plasma appears again. The ‘positive column’ has a longer length and larger volume than the ‘negative glow’. Finally, the ‘anode spot’ is located on the cylinder surface, which is like a lightened blue flame. The luminous and nonluminous regions of the discharge image are similar to those observed in [24,25]. Therefore, the discharge is an atmospheric pressure glow discharge (APGD). 

**Figure 11:**
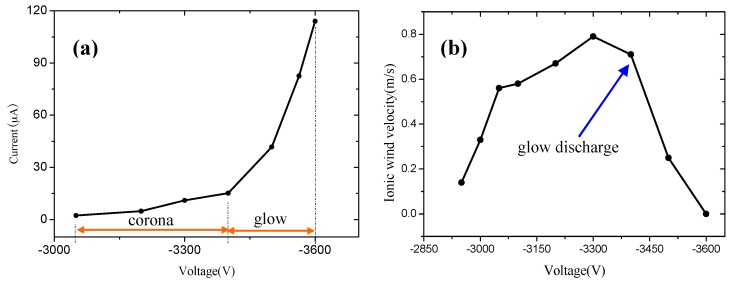
The discharge properties of the chip. (**a**) The volt-ampere characteristics; and (**b**) ionic wind velocities as a function of applied voltage.

Figure 11a shows the volt-ampere characteristics of the chip. As can be seen from the figure, the discharge current is about 2 μA–15 μA in the corona discharge. With the applied voltage beyond −3500 V, the corona discharge transitions to the glow discharge and the discharge current increases significantly. As the arc discharge is harmful to the microneedle and test instruments, it does not increase again when the glow discharge appears.

From the above analysis, it can be seen that there are corona discharge, glow discharge, and arc discharge phases. To compare the ionic wind velocities at different discharge phases, an experiment was conducted. The ionic wind velocity is measured by using an anemometer (Testo 405-V1) with range and sensitivity of 0–10 m/s and 0.01 m/s, respectively. The velocities were measured at a distance of 1 mm from the outlet of the chip. 

Figure 11b shows that the ionic wind velocity is up to 0.14 m/s when the applied voltage reaches −2940 V. At this stage, the corona discharge is ignited and the ionic wind appears. With increasing applied voltage, the ionic wind velocity increases correspondingly until reaching 0.79 m/s when the glow discharge appears. After that, the ionic wind velocity decreases with increasing applied voltage in the glow discharge phase. At the end of the corona discharge, a maximum ionic wind velocity of 0.79 m/s is obtained at a voltage of −3300 V. When the applied voltage reaches −3600 V, the discharge current is larger and the ionic wind velocity is almost close to zero. This result is also observed in our previous research [17]. This shows that the maximum ionic wind velocity appears during the transition from corona discharge to glow discharge. To achieve maximum velocity, the glow discharge should be avoided. 

### 3.2. The Sample Injection and Ionization

According to the above analysis, the maximum ionic wind velocity is achieved when the applied voltage reaches −3300 V. The other experimental conditions are the same. 

**Figure 12:**
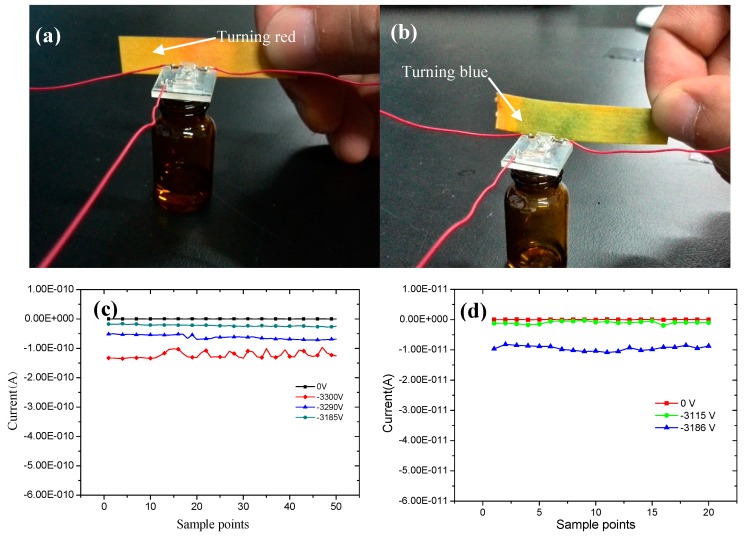
The sample injection and ionization experimental results: (**a**) acid injection; (**b**) ammonia water injection; (**c**) acid ionization current; and (**d**) ammonia water ionization current.

A 0.5 mL amount of acetic acid and 0.5 mL of ammonia water are poured into a 3 mL box separately. In the experiment, the hole on the chip is over the small box. The pH paper wetted with clear water is placed in front of the chip outlet. When the applied voltage is switched off, the color of the test paper will not change. From Figure 12 it appears that pH paper turns red and blue in the presence of acetic acid and ammonia water, respectively. Thus, this proves that the samples have been injected through the chip. 

The ionization experiment was conductedby using a Faraday cup, as shown in Figure 6. When the sample is ionized by the chip, the ions collide with the Faraday cup and are converted into current. When the Keithley 6487 unit was connected to the computer, the measured current data is stored and handled by the LabVIEW software. With a sample frequency of 0.5 Hz, the current data for acid and ammonia water is shown in Figure 12c,d. The sample points in Figure 12 represent the experimental point numbers during the experiment.

When the applied voltage is 0 V, there is no current detected, and no discharge phenomenon. As the applied voltage increases to −3185 V, there is a current of approximately −10 pA detected. With an increase in applied voltage, the current also increases correspondingly. When the applied voltage increases to −3300 V, the current increases to −150 pA. A similar situationarose with ammonia water. In this condition, the cuurent is about −10 pA at an applied voltage of −3186 V.

## 4. Conclusions

In this paper, we have developed a needle-to-cylinder electrode chip based on LIGA technology. The needle tip is 20 μm in diameter and 10 μm in thickness. The whole dimension of the chip is 11 mm × 10 mm × 2.3 mm. Without an air supply and at room temperature, corona discharge is realized when a voltage of −3050 V is applied between the needle and the cylinder electrode. The Trichel pulse appearing on the oscilloscope proves the existance of corona discharge. With an increase in the voltage, corona discharge transits to the glow discharge. The Trichel turns into a DC line when the applied voltage is −3563 V, which means that glow discharge appears. The images recorded by a Nikon camera also show that there is stable corona and glow discharge. We have experimentally demonstrated that the chip yields an ionic wind based on the air discharge. The interesting result is that the ionic wind velocity increases from 0.14 m/s to 0.79 m/s in the process of corona discharge, increasing with the applied voltage. However, the ionic wind decreases when the corona discharge transitions to glow discharge. The ionic wind velocity is almost zero when the arc discharge appears. The acetic acid and ammonia water sample injection are proved by the pH paper turning red and blue. At the same time, the ionization current reaches several pA. This shows that the chip functions doubly as the air pump and ion source, which can be well applied to analytical instruments, such as IMS, FAIMS, and MS.

## Figures and Tables

**Table 1:** The geometric dimensions of the chip.

Features	Dimensions	Features	Dimensions
Curvature radius of pin	10 μm	Thickness of needle	20 μm
Radius of cylinder	0.2 mm	Thickness of cylinder	1 mm
Center distance between the cylinders	0.9 mm	Inter-electrode spacing between two array cylinders	3 mm
Radius of injection hole	0.75 mm	Thickness of substrate of pin electrode	0.49 mm
Height of top substrate	0.5 mm	Length of pin electrode	3 mm
Height of bottom substrate	0.8 mm	The whole dimension of the chip	11 mm × 10 mm × 2.3 mm
